# Physical activity and sedentary behavior among ambulatory children with cerebral palsy using accelerometer: a cross-sectional study

**DOI:** 10.3389/fped.2024.1463288

**Published:** 2024-09-19

**Authors:** Njoud Aydhah Alamoudi, Maha F. Algabbani, Muhammad O. Al-Heizan, Adel A. Alhusaini

**Affiliations:** Department of Health Rehabilitation Sciences, College of Applied Medical Sciences, King Saud University, Riyadh, Saudi Arabia

**Keywords:** accelerometer, physical activity, children, sedentary behavior, cerebral palsy

## Abstract

**Background and objective:**

Physical activity (PA) is paramount for childhood development and growth. However, children diagnosed with Cerebral Palsy (CP) were often considered sedentary, and their physical inactivity was associated with adverse health conditions and complications. Therefore, this study aimed to objectively describe and compare the PA levels and SB levels of children with and without CP of the same age group. It also studied the factors correlating with PA, SB, and step count per day in children with CP.

**Subjects and methods:**

A cross-sectional study using a wrist-worn accelerometer was conducted. PA and SB were measured over seven consecutive days.

**Results:**

Eighty-five children aged 6–12 years, consisting of 41 children with CP and 44 TD children, participated in this study with a mean age of 9.18 ± 1.95 and 8.45 ± 1.78 years, respectively. According to the gross functional measures, 53.6% of children with CP were classified as first classification. A significant amount of time was spent in SB and Light PA (LPA) by children with CP compared to TD children, and no significant differences were observed in moderate PA (MPA) or step count. Gender mainly affected MPA as girls spent more time in MPA than boys. The age, height, and weight of children with CP correlate significantly with SB. As children's age, height, and weight increase, SB increases. Additionally, children with higher weights have lower step counts per day.

**Conclusion:**

This study showed that children with CP spend more time in LPA and SB than typically developed children. Therefore, concerted efforts are needed to encourage physical activity and reduce the sedentary lifestyle, to take into account the gender and anthropometric measures of children to enhance the quality of life among children with CP, and to consider gender and anthropometric measures of the children.

## 1 Introduction

Cerebral palsy (CP) is a neurological disorder that affects movement and coordination, resulting from damage or abnormalities to the central nervous system, usually occurring before, during, or shortly after birth (1, 2). It is considered one of the most common motor disabilities among children. CP types include spastic, dyskinetic, ataxic, and mixed CP (1, 2). Moreover, Children with CP can be classified according to their functional level using the Gross Motor Function Classification System (GMFCS) (3).

Children with CP may experience difficulty with motor skills, balance, and posture, which may have an adverse effect on their overall health and well-being (4, 5). Regular physical activity (PA) is one aspect of maintaining a healthy lifestyle for children with CP. PA is crucial in enhancing physical fitness, promoting muscular strength, improving flexibility, sleeping quality, and reducing the risk of obesity and other health issues (6–9). Moreover, the negative consequences of inactivity and sedentary lifestyles are already evident during the developmental ages (10, 11) and continue into adulthood (12, 13). In addition, inactivity may lead to low self-esteem and lower academic achievement among school-aged children and adolescents (5–17 years of age) (14, 15). However, children with CP may face unique challenges when participating in physical activities (16, 17).

Physical activity (PA) is “any form of skeletal muscle movement that results in energy expenditure” (18). A moderate to vigorous physical activity (MVPA) of at least 60 min a day is highly recommended by the World Health Organization (WHO) for children between the ages of 5 and 17 (19). Based on energy expenditure, physical activity is divided into three categories: (1) Light Physical Activity (LPA), (2) Moderate Physical Activity (MPA), and (3) Vigorous Physical Activity (VPA). Sedentary behavior (SB) refers to waking behaviors characterized by a lower energy expenditure than 1.5 METS (physical intensity units) during sitting, reclining, or lying (19). Further, several step-count guidelines recommend that children walk 12,000 steps daily (20). This recommendation emphasizes the need for children to move regularly throughout their day.

According to a study by Molina-Cantero et al. in Spain, only 17.6% of children with CP meet physical activity recommendations that decrease with age and are not affected by gender (21). Another study conducted in Ireland showed that ambulant children with CP (GMFCS I-III) aged 6–10 years spent more sedentary time and less time in PA than their TD children (22). Further, Obeid et al. reported that ambulant youth with CP aged 8–17 years in Canada spend more sedentary time than their peers (23). In another study, Janzen et al. examined the PA levels of ambulatory children and adolescents with CP and their typically developing peers, finding that individuals with CP had lower MVPA than those without CP (24). According to Ginis et al., people with disabilities are 16%–62% less likely to meet prescribed PA levels and are more likely to develop health problems (25). Despite the well-documented health benefits of performing daily physical activity, there needs to be more data allowing us to determine the child's level of physical activity in Saudi Arabia.

A recent study conducted by Alghamdi and Alsaigh to evaluate the physical activity level among children with disability in Saudi based on an online survey showed that there is less physical activity among children with disabilities compared to their peers who are typically developing (26). The most available data on large-scale physical activity assessments are based on self-reported measures, a method with validity and reliability issues. In addition, all studies focused on typically developing children (27), while no studies have been conducted on children with CP. Objective measures of physical activity may provide valid and reliable estimates of physical activity and are available for use in large-scale assessments of physical activity (28).

Regular monitoring of physical activity levels and sedentary behavior in children is crucial to provide valuable insights into children's overall health and well-being. It helps to identify trends, patterns, and disparities among different groups of children, such as socioeconomic status, gender, or clinical characteristics. It is important to answer the following questions: What is the level of physical activity and sedentary behavior in children with cerebral palsy? What is the difference between physical activity and sedentary behavior in children with and without cerebral palsy? What factors affect PA, SB, and step count in cerebral palsy children? We hypothesized that the level of physical activity and sedentary behavior in children with CP would be significantly lower than that of TD children. This information can be used to develop targeted interventions and strategies to address the specific needs of children and ensure equal access to physical activity opportunities. Therefore, this study aimed to objectively describe and compare LPA, MPA, VPA, and SB in children with and without CP aged 6–12 years old and to study the factors that correlate with PA, SB, and step count per day in children with cerebral palsy.

## 2 Methods

### 2.1 Study design, setting and sampling methods

A cross-sectional quantitative descriptive study was conducted in this study. Children with CP were voluntarily recruited from the rehabilitation departments at the Disabled Children Association (DCA) and the Prince Sultan Military Medical City (PSMMC) in Riyadh, Saudi Arabia. The in-charge therapist in rehabilitation departments provided a list of eligible children. The principal investigator contacted parents of eligible children by phone or email with an invitation letter. Children with CP were recruited using a convenience sample (Participants were selected based on their accessibility or availability. The TD group was recruited using a snowball sampling method (Recruitment occurs by asking existing participants for recommendations of additional participants who meet the same criteria.). The study was conducted from September 2020 to February 2021.

### 2.2 Institutional review board statement

Permission for the study was granted by the research ethics committee in King Saud University (E-19-4520) in the 3rd of February 2020 and Prince Sultan Medical Military City (PSMMC) (HP-01-R079) in the 14th of September, 2020, and Permission for the study was granted by the Disabled Children’s Association (DCA).

### 2.3 Informed consent statement

The participants’ parents were given a detailed explanation of the study protocol. Once all questions had been answered, the parents were satisfied. We obtained written consent forms from the parents and legal guardians of the children and assent forms from the participants before beginning the study.

### 2.4 Participants

The study included children with CP who were: (1) aged 6–12 years old, (2) diagnosed with spastic cerebral palsy, and (3) had a GMFCS level of I: Walk without limitations but with some difficulty with balance and coordination, II: walk independently but may require assistive devices such as crutches or walkers, or III: Can walk with assistive devices but may need more support and assistance (3). Children who had Botox injections for lower extremities within the last three months. Children with recent surgical interventions for lower extremities, such as soft tissue releases within six months, had vision impairment, had an uncontrolled seizure, had a musculoskeletal injury, had cardiopulmonary disease, or were unable to follow simple instructions were excluded. The study included typically developing children ages 6–12 (without visual impairment, neurological condition, musculoskeletal injury, cardiopulmonary disease, or recent trauma) of the same age group as the CP children.

### 2.5 Sample size

The sample size was calculated using power analysis software (G*Power, version 3.1.9.4) (29). Using a one-tailed test for the difference between two independent groups, with a large effect size (d = 0.8) (22, 30), α=0.05, and a power of 0.95, a total sample of 70 participants with two equal-sized groups of *n* = 35 was required. In addition, a 20% estimated dropout rate was added.

### 2.6 Data collection

Data were collected by the same principal investigator in all cases.

#### 2.6.1 Characteristics of sociographic, anthropometry, and clinical characteristics of participants

The parents filled out a data collection sheet to report sociodemographic data. Typically developing children and children with CP with no skeletal deformities were measured by standing against a wall-mounted tape measure. When skeletal deformities exist, such as flexion deformities of the lower legs (29), height is estimated using segmental measurements based on the knee height equation, where height (cm) = [2.69 × knee height (cm)] + 24.2 (30). The height was measured to the nearest 0.1 cm. Using a mechanical scale (Detecto balance beam scale) (31–33), the child's weight was measured to the nearest 0.1 kg while barefoot. A mechanical chair scale was used to measure the weight of those children with GMFCS level III (33). Body mass index (BMI) was calculated according to the following formula:

$$\mathrm{BMI}=\frac{\mathrm{Weight}\;(\mathrm{kg})}{\mathrm{Height}\;\mathrm{squared}(m^{2})}$$

BMI percentile was measured using an online calculator on the website https://www.cdc.gov. The child's height, weight, sex, Georgian measurement date, and Georgian birth date (34). Clinical information was obtained from the parents and from the medical file.

#### 2.6.2 Physical activity levels, sedentary behaviour and step count per day

Accelerometer model wGT3X-BT (ActiGraph, Pensacola, FL, 2013) was used to measure children's PA and SB objectively (35, 36). ActiGraph is a small device (4.6 cm×3.3 cm×1.5 cm), lightweight (19 grams), and battery-powered. The motion sensor was designed to detect accelerations in 3 axes [horizontal (x), vertical (y), and Perpendicular (z)]; it could record acceleration data at rates ranging from 30 to 100 Hertz and store it in epoch lengths from 1 to 240 s (37–39). Using ActiLife 6 Data Analysis Software, participants’ height, weight, gender, and date of birth were recorded. A 60-second epoch-length file was used to calculate PA (LPA, MPA, and VPA) and SB times in minutes from raw data collected by the accelerometer. Additionally, the step count was recorded and used as an indicator of PA level (40).

### 2.7 Procedures

To obtain the most valid datasets, the accelerometer was instructed to be worn for seven days (school days were five and weekends were two) (37) and at the same time of year (middle of academic year). Considering that the accelerometer is not water-resistant, it was instructed to be removed only when the child is exposed to water (such as when washing or swimming) (37). For the accelerometer dataset included in this study, a minimum wear time of 8 h (480 min) per day for four days, including a weekend, was deemed valid, as this met the criteria for inclusion in the International Children's Accelerometry Database (41).

A standardized procedure was used to initialize the ActiGraph before it was given to a child. This initialization was conducted with the child's code number, sex, height, weight, date of birth, and race; “Limb” was set to the wrist, and “Side” to the non-dominant hand (42). The monitor was processed in an epoch length of 10 s to capture very short bouts of movement due to the sporadic activity of participants (29, 37, 43). Also, short epoch lengths increased the resolution of the measurement (44). For increased compliance, a strap was used to secure the device around each participant's non-dominant wrist, proximal to their ulnar and radial styloid (45, 46). Accelerometers worn on the wrist are more sensitive to upper body movement, which is considered to be a significant component of children's physical activity [Fairclough et al., (45)].

After receiving the accelerometer and activity diary one week later, the principal investigator downloaded the ActiGraph data from the sensor monitor using the ActiLife software and stored it in the file for further analysis.

### 2.8 Statistical analysis

Statistical Package for Social Studies (SPSS) Version 29 for Windows (IBM SPSS, Armonk, NY, USA) was used for analysis. Shapiro-Wilk tests were used to determine whether the data distribution was normal. For normally distributed continuous data, means and standard deviations are presented, whereas, for non-normally distributed continuous data, medians and interquartile ranges are presented. Data from categorical variables are described using frequencies and percentages.

In order to assess differences between the CP and the TD groups in sociodemographic and anthropometric variables, the Mann-Whitney *U*-test or independent sample *t*-test for continuous variables were applied according to the distribution of data. The chi-square test was applied to categorical variables (Fisher's exact test if the count is less than five).

This study utilized a two-way multivariate analysis of covariance (MANCOVA) to examine the effects of group, gender, and the interaction of group with gender on the variables PA, SB, and step count with age as a covariate.

The relationship of PA, SB, and step count per day with the sociodemographic, anthropometric, and clinical characteristics of children in CP group variables were studied using Superman's rank correlation (for continuous data) or the Eta test (nominal with continuous data). Correlation interrupted as no or very weak correlation, <.1; weak,.1–.3; moderate,.4–.6; strong,.7–.8; and a perfect correlation = 1 (47).

## 3 Results

**Figure 1:**
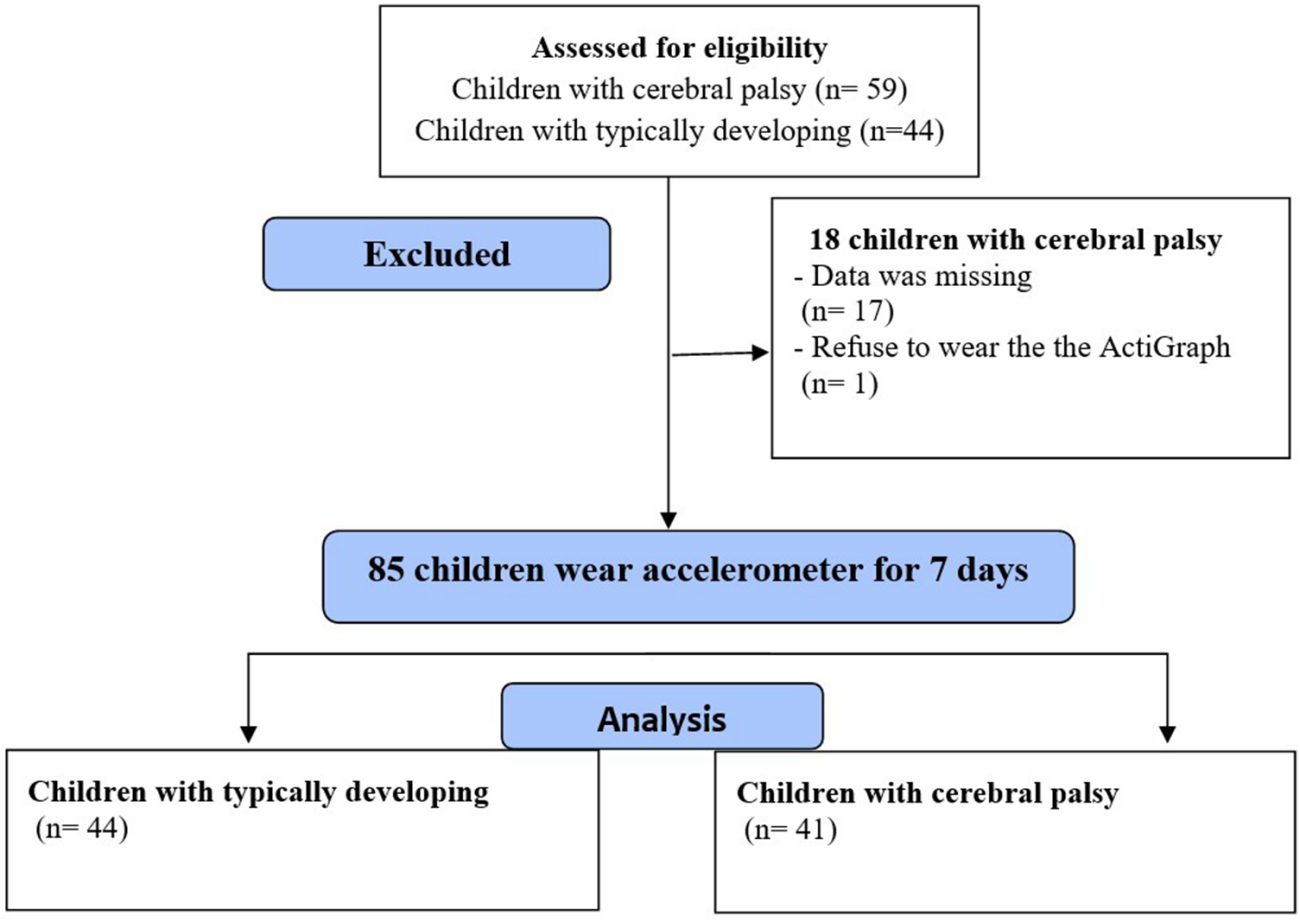
The flow diagram of the participants.

Out of the 103 participants recruited for this study, Data were not available for 17 children, and one child with CP refused to wear the ActiGraph (The flow diagram is shown in Figure 1). This analysis included 85 children, 41 with CP (20 males) and 44 with TD (20 males), with a mean age of 8.83 ± 1.90 years old; all children were enrolled in school. The height data were normally distributed; otherwise, all continuous variables were not normally distributed. All PA scores and step counts were normally distributed.

### 3.1 Characteristics of the participants

Table 1 shows the sociodemographic and anthropometric of the participants as well as the differences between the two groups. In spite of no significant differences in age or gender (*p* = .11, *p* = .76, respectively), typically developing children had higher heights, weights, and BMI scores (*P* < .05). None of the children were involved in a regular physical activity program. Vehicles were used by both groups for the main transportation with no significant differences. A significant number of children with CP lived on the ground floor compared to children with TD. The education level of mothers of children with CP was a significantly lower than of mothers of TD children (*p* = .02). while the education level of fathers was not significantly different (*p* = .59). Families’ incomes did not differ significantly between the two groups.

**Table 1:** Participants’ sociodemographic and anthropometric characteristics and the statistical differences between CP group and TD group.

Variable		CP group *N* = 41	TD group *N* = 44	*p* value*
Age (years) Median (1st^,^ 3rd quartiles)		8.44 (6.78, 9.48)	9.19 (7.15, 10.79)	.11^a^
Height in cm Mean ± SD		121 ± 57	131.83 ± 13.8	<.001^b^
Weight in kg Median (1st^,^ 3rd quartiles)		20.00 (18,25)	28.50 (21.00, 39)	<.001^a^
Body mass index in kg/m^2^ Median (1st^,^ 3rd quartiles)		15.12 (14.16, 16,37)	16.80 (14.08, 20,38)	.04^a^
Gender *N* (%)	Boys	20 (48.8%)	20 (45.5%)	.76^c^
	Girls	21 (51.2%)	24 (54.5%)	
Transportation methods *N* (%)	Vehicle transportation	39 (95.1%)	42 (95.5%)	.93^d^
	Pedestrian transportation	2 (4.9)	2 (4.5%)	
Accommodation level *N* (%)	Ground floor	37 (90.2%)	26 (59.1%)	<.001^d^
	first or second floor	4 (9.8%)	18 (40.9%)	
Mother's education *N* (%)	High school or less	26 (63.4%)	17 (38.6%)	.02^c^
	Bachelor or higher	15 (36.6%)	27 (61.4%)	
Father's education *N* (%)	High school or less	22 (53.7%)	21 (47.7%)	.59^c^
	Bachelor or higher	19 (46.3%)	23 (52.3%)	
Income per month *N* (%)	<9,000 SR	23 (56.1%)	26 (59.1%)	.78^c^
	>9,000 SR	18 (43.9%)	18 (40.9%)	

N, number of participants; CP, cerebral palsy; TD, typically developing; cm, centimetre; kg, kilogram;%, percentage.

^a^ Mann–Whitney *U*-test.

^b^ Independent *t*-test.

^c^ Person Chi-square test.

^d^ Fisher's exact test.

* Significant at level *p* < .05.

### 3.2 Clinical characteristic of children with CP

The clinical characteristics of children with CP were: 22 children (53.7%) were classified as GMFCS level I, seven children (17.1%) classified as GMFCS level II, and 12 (29.3%) children were classified as GMFCS III. Among children with CP, 12 (29.4%) were hemiplegic, 27 (65.9%) were diaplegic, and two (4.9%) were quadriplegic. Twelve (29.3%) use a walker, and 29 (70.7%) walk independently. There are 17 (41.5%) individuals who wear only medical shoes and 24 (58.5%) individuals who wear medical shoes with different AFOs and heel raises.

### 3.3 Physical activity (PA) data in TD and CP children

Both children with CP and children with TD wore the ActiGraph wGT3X-BT for a median time of seven days and for a mean duration of 6.9 ± 0.7 h and 6.9 ± 0.4 h per day, respectively. 6 (14.6%) of children with CP and 7 (15.9%) of the TD children exceeded the recommended step count per day, but none of the children reported any VPA, meaning that none met the recommended PA level. Table 2 shows descriptive data for PA, SB, and step count. The MANCOVA test revealed that there was a main effect of group in SB and LPA (children with CP spent significant more time in the SB and LPA), and there was a main effect of gender on MVA where girls spent more time in MPA than boys. There was no interaction between group and gender on physical activity, sedentary behavior, and step count (Table 3).

**Table 2:** Descriptive statistics for PA variables.

Variables	CP group			TD group			Total	
	Girls *N* = 21	Boys *N* = 20	Total *N* = 41	Girls *N* = 24	Boys *N* = 20	Total *N* = 44	Girls *N* = 45	Boys *N* = 40
SB (min/day)	830 ± 90	847 ± 139	838 ± 115	770 ± 82	826 ± 91	796 ± 90	798 ± 90	836 ± 116
LPA (min/day)	78 ± 17	80 ± 18	79 ± 17	69 ± 8	66 ± 7	67 ± 8	73 ± 14	73 ± 15
MPA (min/day)	379 ± 87	361 ± 106	370 ± 96	394 ± 86	331 ± 82	365 ± 89	387 ± 85	346 ± 95
Step count per day	8,929 ± 2,917	9,406 ± 2,884	9,162 ± 2,875	9,504 ± 2,433	9,176 ± 2,693	9,355 ± 2,529	9,236 ± 2,655	9,291 ± 2,757

Data presented as mean ± standard deviation. SB, sedentary behaviour; LPA, light physical activity; MPA, moderate physical activity; CP, children with Cerebral palsy; TD, typically developing children; N, number of participants.

**Table 3:** The MANCOVA for the main effects of group and gender and the interaction of group and gender on physical activity, sedentary behavior and step count.

Source	Dependent variable	df	F	Sig.	pη2	95% CI for difference		Observed power
						Lower bound	Upper bound	
Group	SB	1	7.657	.007	.09	16	99	.78
	LPA	1	15.477	<.001	.162	6	17	.97
	MPA	1	.154	.696	.002	−44	30	.06
	Step count per day	1	.719	.399	.009	−1,654	666	.13
Gender	SB		3.525	.064	.042	−79	2	.46
	LPA		.010	.921	.000	−5	6	.05
	MPA		5.365	.023	.063	6	78	.63
	Step count per day		.005	.943	.000	−1,177	1,095	.05
Group × Gender	SB		.399	.530	.005	–	–	.09
	LPA		1.086	.301	.013	–	–	.18
	MPA		.861	.356	.011	–	–	.15
	Step count per day		.246	.622	.003	–	–	.08

SB, sedentary behaviour; LPA, light physical activity; MPA, moderate physical activity; df, degrees of freedom, F, F-value, Sig., significance level (<.05); pη^2^, partial eta-squared; CI, confidence interval.

### 3.4 The correlation of PA, SB, and step count with sociodemographic, anthropometric, and clinical characteristic in children with cerebral palsy

Eta test showed very weak correlation of SB, LPA, MPA, and step count per day with gender (Eta = .08,.07,.09,.08, respectively) and very weak to weak correlation with type of CP (Eta = .09,.20,.13,.31, respectively) and very week correlation with type of walking aids (Eta = .01,.14,.01,.12, respectively). On the other hand, Table 4 shows the other variables. Age, height, and weight moderately positively correlate with SB and negatively with MPA, indicating that the SB increases and MPA decreases with an increase in the children's age, height, and weight. No other correlation with the other variables was observed (Table 4).

**Table 4:** Correlation of PA, SB, and step count with sociodemographic, anthropometric, and clinical characteristic in children with cerebral palsy.

Variables	SB	LPA	MPA	Step count per day
Age (years)	.43**	.02	−.44**	−.12
Height in cm	.49**	.06	−40**	−.29
Weight in kg	.44**	−.09	−.44**	−.37**
Body mass index in kg/m^2^	.19	.04	−.16	−.15
GMFCS	−.19	.05	.03	−.18
Accommodation level	.01	−.03	−.11	−.04
Mother's education	−.08	.24	.06	.03
Father's education	.12	.12	−.08	−.02
Income per month	.09	.00	.08	.20

SB, sedentary behaviour; LPA, light physical activity; MPA, moderate physical activity; cm, centimetre; Kg, kilogram; m^2^, meter square; GMFCS, gross motor functional classifications.

** Significant differences *P* < .001 based on Spearman's rank correlation.

## 4 Discussion

Children with and without CP aged 6–12 were evaluated objectively for PA level and SB. A comparison between their results supported our hypothesis that children with CP spend less time in physical activity.

In this study, children with and without CP did not meet the recommended levels of physical activity, spending most of their time on SB, and only a small percentage achieved the recommended daily step count. These results were consistent with other national (48–50) and international (51–53) studies that reported that the majority of children with and without disability fail to meet the recommended physical activity level. Many factors can explain the persistence of lower physical activity worldwide. The increasing prevalence of sedentary lifestyles and reliance on electronic devices may contributed to the lack of physical activity among children (54). Moreover, Parental engagement and support also play a crucial role in ensuring that children meet the recommended physical activity level (55). Many parents fail to prioritize physical activity in their children's lives, either due to work commitments, lack of time, or misconceptions regarding its importance. The social and cultural context in which children grow up can also affect their physical activity levels (56).

A significant amount of time was spent in SB and LPA by children with CP compared to TD children, and no significant differences were observed in MPA or step count. The results of this study are in agreement with previous international studies that used accelerometry to compare the PA between children with and without CP (22, 57–59). In the literature, several studies found that children with CP spent more time in sedentary behavior compared to typically developed children (22, 23, 60). A recent study by Okur et al. (59) compared physical activity using an accelerometer and activity diary in a sample of 48 children, including 24 children with CP level I-II and 24 typically developed children aged 6–18 years, and they found that the CP (I-II) spent less time in moderate PA than typically developed children. This study, however, found no significant differences between MPA levels in the two groups of children. Low levels of PA in children with CP may be due to the limitations imposed by their condition, such as difficulties with mobility or balance. While no significant differences were observed in MPA or step count between the two groups, this does not necessarily mean that children with CP engage in the same level of moderate-intensity physical activity as their TD peers; it may be because TD children's MPA levels are declining upstream. Various factors may also have to be taken into account, such as motivational differences and access to appropriate physical activities. Other Possible explanations for this may be that the study was conducted during the pandemic coronavirus (COVID-19) lockdown (social isolation, schools, and clinics closed); this may have an impact on the typically developed children's activity (61). Moreover, the results agree with previous international studies that used physical activity questionnaires to compare the PA between children with and without CP (62, 63).

The children with CP had less PA, maybe because the caregivers commonly thought that children with CP were fragile; therefore, they may be overprotective to prevent possible injuries (64). In addition, the researcher thought that children with CP attempt to engage in physical activity, but their significant developmental problems, such as balance disturbances, spasticity, and general weakness, might prevent or limit them from participating in physical activity (33, 65).

Some studies revealed both groups (children with CP and TD) have similar PA levels (58, 66). For example, a study by Bjornson et al. using a Step-Watch monitor found that children with CP in GMFCS level I spent a similar amount of PA as the TD children (58). Stevens et al. found no difference in the number of daily steps between children with CP (GMFCS level I-II) and TD children aged 4–10 years by using an accelerometer (66). An explanation for that might be that the sample of children with CP who have the highest functioning level, classified in GMFCS levels I-II, walk and tend to be more similar to TD children.

Also, in the current study, the TD children were more overweight than children with CP, which is consistent with accumulating evidence that reported that increased time spent in sedentary behavior is associated with overweight and metabolic dysfunction in children with typical development (67, 68). Another explanation may be that families of children with CP are more sensitive to the adverse effects of sedentary behaviors (SB) by maintaining the home exercise program of physiotherapy (69).

Furthermore, vigorous physical activity in this study appeared to be non-existent, which is consistent with previous studies on people with intellectual disabilities (70–72). This may be due to the fact that children with CP are unable to achieve the endurance and velocity required for vigorous activities (59); due to the presence of spasticity, the muscle's physiological changes, such as reducing muscle volume and poor muscle growth, lead to poor force production (73, 74), then lack of ability to activate maximal activity in the muscle needed in the vigorous activity (75).

Gender had a main effect on MPA in this study. It was interesting to find that girls spent more time in MPA than boys which was contradicted by other previous studies (76, 77). It is possible that a significant motivator for engaging in physical activity is body image concerns. Research suggests that girls tend to be more affected than boys by body image concerns (78). Due to this self-consciousness about their appearance, they may be less motivated to engage in physical activity.

The anthropometric measures of children with CP correlated significantly with SB. As children's age, height, and weight increase, SB increases. Additionally, children with higher weights have lower step counts per day. Firstly, age was a factor that can influence SB patterns. As children age, they may develop preferences and habits that increase their sedentary time, and this finding was in line with other studies for children with and without CP (79–81). This can be a result of spending more time watching television, playing video games, or engaging in other forms of screen-based activities. These activities can provide entertainment and relaxation, but they also contribute to increased SB. Height and weight (usually associated with each other) were other factors that can influence SB. Similar to children without disability, children with increased weight have a higher likelihood of engaging in sedentary behaviors (82, 83). Unfortunately, there is a lack of studies exploring the association between anthropometric measures and PA levels for children with CP to compare with.

Surprisingly, the clinical characteristics of children with CP, such as the type of CP and GMFCS level, did not correlate with PA, SB, or step count per day, unlike in previous studies (21, 84). It is suggested that other factors that have not been examine in this study CP may directly influence their ability to participate in physical activity such as cognitive ability, motivations, and parental support and encouragement specially (16, 85).

The significantly lower PA levels in children with CP than in TD children highlight the importance of addressing the PA to prevent chronic disease. Study findings may inform programs designed to enhance PA and decrease SB among both CP and TD children. To encourage increased physical activity and reduce sedentary behaviors in this population, public health planners need to know PA levels in this population (86).

A study of children with CP's physical activity levels is essential for assessing their overall health, promoting physical activity, and developing intervention strategies. While there are challenges, such as variability and limited reliable assessment tools, the use of objective measuring tools in this study for Saudi children with cerebral palsy provides valuable data that enhances the accuracy of activity level assessment. It provides a consistent, quantifiable metric that minimizes subjective errors. Thus, more tailored and effective intervention strategies can be developed, improving the overall health outcomes of these children.

As a result of using an objective measure (accelerometer), this study offers several strengths, including reliability, validity, and quantitative data, but it also has some limitations that should be taken into account when interpreting the findings. The sample may not be representative of all Saudi children; it was limited to Riyadh city. To get more insight and accuracy of results and comparisons, future studies need to use a larger sample size that includes GMFCS VI-V and different ages. In addition, future data collection could include details of school hours for children with CP and TD. Another important limitation is that this study was conducted during the COVID-19 pandemic, which could influence the results.

Future studies should focus on how physical activity can enhance gross motor proficiency in children with disabilities. By targeting specific exercises and activities, researchers can identify the most effective methods to improve motor skills. This could lead to better-designed programs that significantly boost the physical capabilities and quality of life for these children.

## 5 Conclusion

This study is the first study conducted in Saudi Arabia that used an objective method to measure the PA and SB among children with CP compared to TD peers. Our study noticed that LPA and SB among children with CP aged 6–12 years were higher than TD children. This is an important finding to be considered by family and healthcare professionals who work with children with CP since children with CP are at risk of developing diseases associated with physical inactivity. Therefore, there is a need for well-designed, preventive health promotion strategies and interventions to promote physical activity and physical fitness levels and improve the quality of life for children with CP.

## Data Availability

The raw data supporting the conclusions of this article will be made available by the authors, without undue reservation.
